# Initial Optimization of the Growth Conditions of GaAs Homo-Epitaxial Layers after Cleaning and Restarting the Molecular Beam Epitaxy Reactor

**DOI:** 10.1021/acsomega.3c04777

**Published:** 2023-08-29

**Authors:** Dawid Jarosz, Marcin Stachowicz, Piotr Krzeminski, Marta Ruszala, Anna Jus, Pawel Sliz, Dariusz Ploch, Michal Marchewka

**Affiliations:** † International Research Centre MagTop, Institute of Physics, Polish Academy of Sciences, Al. Lotnikow 32/46, PL-02-668 Warsaw, Poland; ‡ Center of Microelectronics and Nanotechnology, University of Rzeszow, Institute of Materials Science and Engineering, Al. Rejtana 16c, PL-35-959 Rzeszow, Poland; § Institute of Physics, Polish Academy of Sciences, Al. Lotnikow 32/46, PL-02-668 Warsaw, Poland

## Abstract

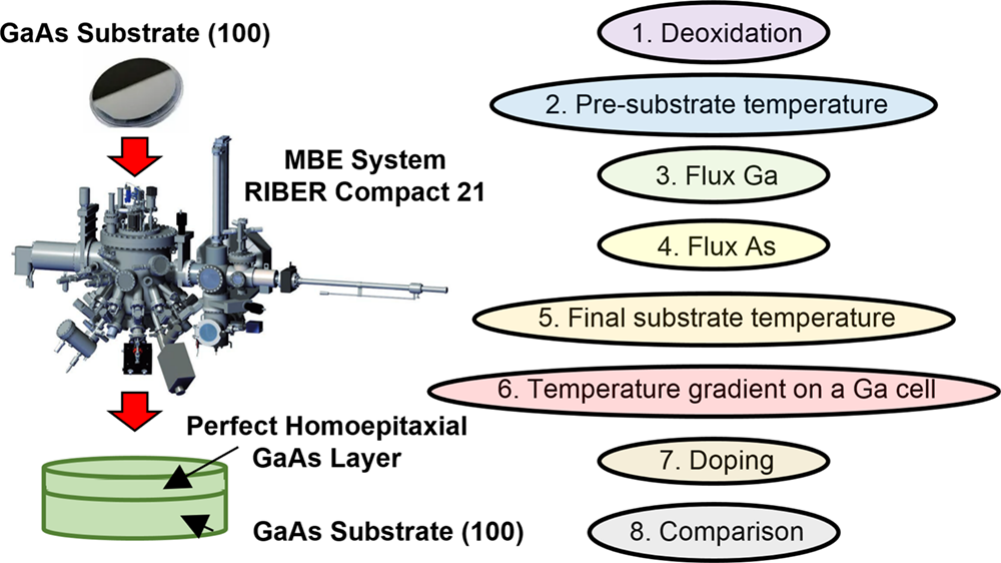

The molecular beam epitaxy (MBE) technique is renowned as the most suitable for the growth of high-quality crystalline materials and nanostructures such as GaAs. However, once established, optimal growth parameters required for repeatability of top-quality structures may be easily lost as MBE is highly sensitive to any changes in the system. Especially, routine servicing procedures, which include any activity which requires unsealing of the growth chamber, are devastating for developed growth parameters and force the necessity of recalibration. In this work, we present the process of growth parameter pre-optimization for obtaining homoepitaxial GaAs layers after servicing and restarting the MBE system. Namely, we present how each step of reestablishing optimal growth condition influences various characteristics of obtained GaAs layers. Those include in situ, structural, and spectral measurement techniques. An additional aspect was to compare the optimal conditions for the growth of homoepitaxial GaAs layers from two growth campaigns in which the main difference is the addition of an ion pump and increasing the temperature gradient on the Ga cell.

## 1 Introduction

Among known and widely utilized growth techniques for crystallization of GaAs-based materials, MBE is the one that is considered the most precise, in terms of growth parameters control such as substrate temperature, element flux ratio, and also sets of available in situ monitoring techniques, which are indispensable for obtaining state-of-art quality crystals. The idea behind the MBE is relatively trivial as it just is delivering only those components which are to be included in the structure on the surface of the supporting crystal. However, practical realization of such an idea required a certain technological advancement, which was achieved in the 1960s by Bell Laboratories’ employees Arthur Jr. and Cho, which successfully evaporated epitaxial GaAs on GaAs substrate.^1,2^ The high dynamical vacuum in the growth chamber enables utilization of various in situ measurement techniques such as diffraction of high energy electrons RHEED, mass spectroscopy, or laser reflectometry. As it was already alluded MBE requires periodic servicing and replacement of worn off elements, refill of effusion materials or cleaning the reactor inside from unwanted deposits accumulated due to number of growth process cycles, which are associated with the cyclical loss of the established optimal growth parameters. Although, the optimal growth processes for obtaining homoepitaxial GaAs layers have been already reported by many research groups, e.g.,^3−7^ those require repetitive determination after each alteration introduced to the MBE system. The high quality of homoepitaxial GaAs is required for e.g. spintronic devices,^8,9^ ultra-height quality two-dimensional electron gas,^10^ an ultrabroadband upconversion device,^11^ or next-generation high-energy particle tracking detectors.^12^

In this work, we present procedure of initial growth parameters determination for obtaining homoepitaxial GaAs layers after MBE maintenance, which may standardize the process and minimize time to achieve the required outcome.

## 2 Methods

As it was mentioned in the Introduction section, the Riber Compact 21 T3-5 reactor underwent the maintenance procedure before the start of the growth cycle. The maintenance included mechanical cleaning of the growth chamber’s interior, refilling ultra-pure materials such as Ga, In, Al to classic ABN60 double-zone effusion cells, and ABN135 dopant cells with Be and GaTe. Refill of the elements was concluded with As and Sb loading into valve cracked VAC 500 and VCOR 300 cells, respectively. The maintenance procedure also included a replacement of all filaments of vacuum gauges and adding a new PI400TTZ ion pump to already the equipped CRYO-TORR 8 pump, which increased the efficiency of the vacuum subsystem. Less invasive but also beneficial improvements were introduced by installing the Bayard Alpert gauge and fluorescent screen for RHEED. After sealing the MBE system, the routine baking procedure was conducted according to the manufacturer’s instructions. Furthermore, all molybdenum substrate holders were refurbished with mechanical, chemical, and thermal cleaning in accordance with instructions provided by the manufacturer. Increasing the pumping efficiency of the MBE reactor by means of a new ion pump significantly changes the optimal growth conditions and has a positive effect on the purity of the deposited layers, which was shown by comparing the growth conditions from two campaigns.

The initial growth procedure of homoepitaxial GaAs, GaAs:Te, and GaAs:Be layer was conducted on one-side polished 350 μm-thick (100) GaAs:Un substrates. The establishment of the growth temperature was realized with infrared pyrometer IRCON, in which the spectral response peak is at 930 nm and the measuring range is between 450 and 1200 °C. Its calibration also was conducted by measurement of the oxygen desorption of GaAs wafer. The RHEED patterns were monitored, with STAIB Instruments NEFRE-10 as the electron source, during the growths. The growth rate was determined by postgrowth comparison of the growth time to the thickness value provided by the DektakXT profilometer. To determine the initial growth conditions for the homoepitaxial GaAs layers, a number of processes were carried out. Obtained samples were characterized with Atomic Force Microscopy INNOVA, Scanning Electron Microscope HELIOS NANOL B650, Optical Microscope with Nomarski interference contrast OLYMPUS DSX1000, High-Resolution X-ray Diffractometer EMPYREAN 3, and a Hall measurement system with van der Pauw configuration Ecopia HMS-5000.

## 3 Results and Discussion

**Figure 1:**
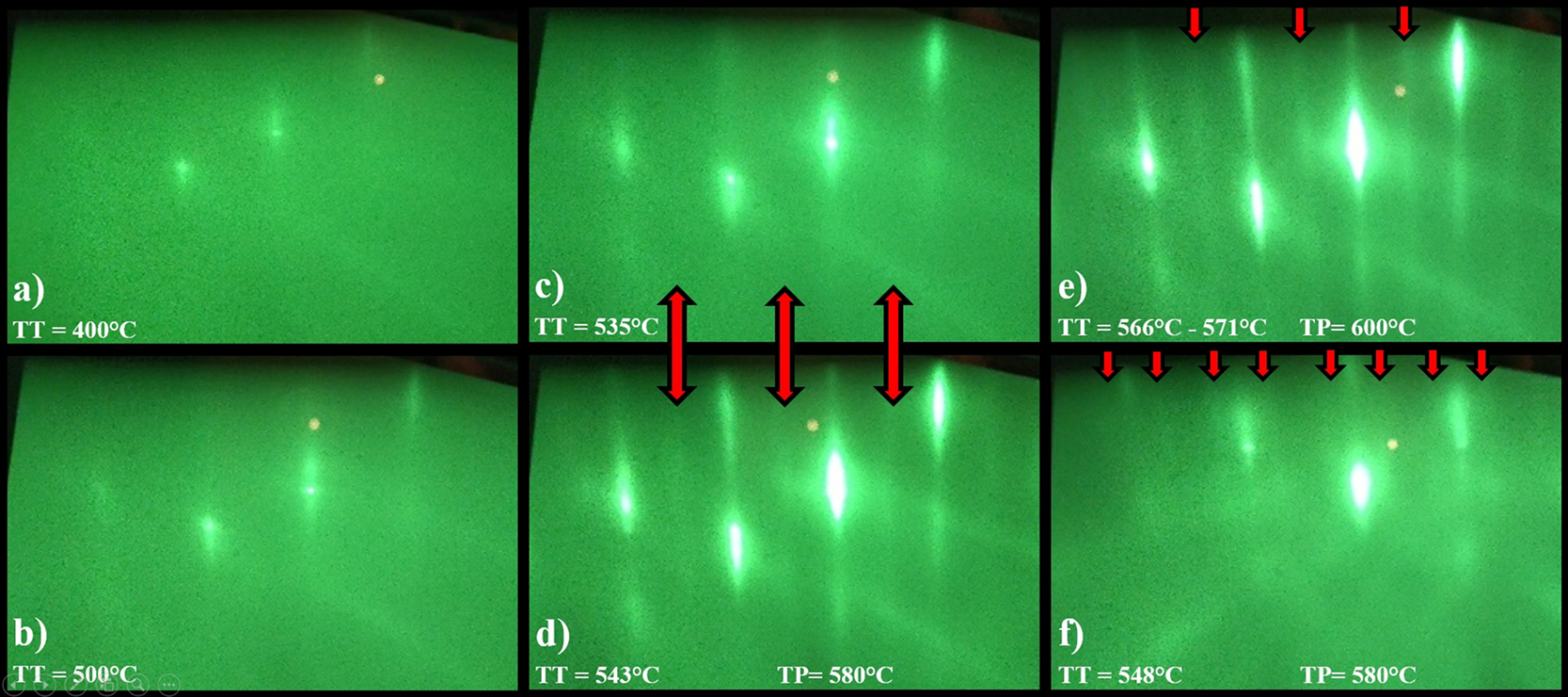
RHEED patterns from GaAs [0–11] substrate during the deoxidation process for temperatures on the main heater, respectively: (a) 400 °C, (b) 500 °C, (c) 535 °C, (d) 543 °C, (e) 571 °C, (f) 548 °C - GaAs [−1–10]. The temperature from the thermocouple on the main heater is shown as TT. The substrate temperature obtained from the calibration pyrometer was designated TP. Red arrows indicate where changes were observed in the RHEED pattern.

The deoxidation process of the GaAs substrate is the initial step of the commencing growth process, and its indication is the evolution of the RHEED pattern which is presented in Figure 1.

Next, establishing the gallium flux pressure values is crucial for achieving arsenic-rich growth conditions with 1 μm/h growth rate of GaAs layers. Its representation is the evolution of the RHEED pattern during GaAs growth, while it shifts from arsenic to gallium rich conditions. Optimization of III/V elements flux ratio was determined by surface morphology of the GaAs layers observation. The investigation of the substrate temperature manipulation within the range of 10 °C also was conducted in terms of influence on GaAs layer crystalline quality. The influence of the temperature gradient in the gallium effusion cell on the number of gallium-derived surface defects was also checked. Its result is concluded with the determination of the doping level to p or n conductivity type dependence on the temperature of Be and GaTe dopant cells, respectively. The last step was to compare the optimal conditions for the growth of GaAs layers from the two campaigns.

### 3.1 Deoxidation of the GaAs Substrate

**Figure 2:**
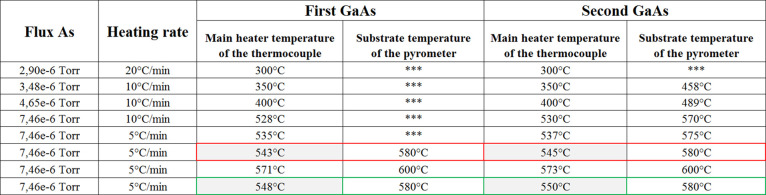
Summary of temperature values for the substrate from the pyrometer and the main heater from the thermocouple for the first 2 deoxidation processes of the GaAs substrate on the same molybdenum holder and for the same values of arsenic flux.

The first growth process was conducted with the arsenic flux level set at about 75% of the maximum capacity of the effusion cell, according to the producer’s documentation, as the working flux reached 7.46 × 10^–6^ Torr, which was enough to ensure arsenic-rich growth conditions. The warming up rates and the final temperature of the main heater together with arsenic flux values are presented in Figure 2.

**Figure 3:**
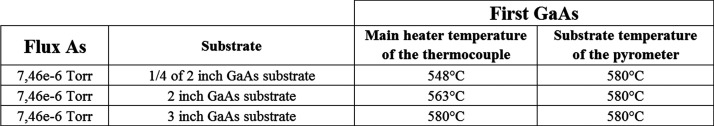
Summary of temperature values for the substrate from the pyrometer and the main heater from the thermocouple for the first three deoxidation processes of the GaAs substrate of different sizes performed at the same arsenic flux values.

As it can be noticed, the warming up rate was reduced to 5 °C/min after reaching 528 °C and above. It is commonly known that GaAs substrates’ surface is covered with the oxygenic layer, and during deoxidation, it is protected with As flux against losing As element and pitting. As the GaAs substrate is thermally processed by warming up to about alluded 528 °C, free As bonds are created which are ready for delivering Ga for the commencing growth process. As the growth is conducted in As-rich conditions, the reconstruction shadows appear in between the main RHEED streaks. The RHEED pattern change was recorded at this point, which is represented in Figure 1d. After occurrence of the RHEED pattern change, the temperature increase was stopped and the pyrometer was calibrated at 580 °C. At this point, the annealing temperature of the substrate was set at 600 °C for another 10 min and cooled down to 580 °C after that time. The change of RHEED pattern in accordance to temperatures was recorded, which is depicted in Figure 1e,f, respectively. The GaAs substrate emissivity after full deoxidation influenced the pyrometer readout which indicated 5 °C lower temperature than before annealing therefore, in order to maintain a constant temperature of the substrate at 580 °C, the temperature of the main furnace was increased by 5 °C. This fact is marked in gray in the background in Figure 2. Moreover, during the second analogous growth process, the same molybdenum substrate holder was utilized, the required temperature of the main heater was recorded which was 2 °C higher in the first process both before and after deoxidation, which is marked as red and green frames in Figure 2, respectively. This points that the molybdenum substrate holder changed after the first growth process and it also influences the pyrometer’s readout due to change of the emissivity. The emissivity changes of the holder in latter growth processes were negligible. The temperature of the main furnace was lower than the temperature of the pyrometer after calibration. This was due to the calibration of the thermocouple on target GaAs substrates of 3 inch size while all processes included in this work were done on 1/4 of 2 inch substrates. A comparison of main heater temperatures as a function of substrate size is shown in Figure 3.

### 3.2 GaAs Growth Rate Dependence on Gallium Flux

**Figure 4:**
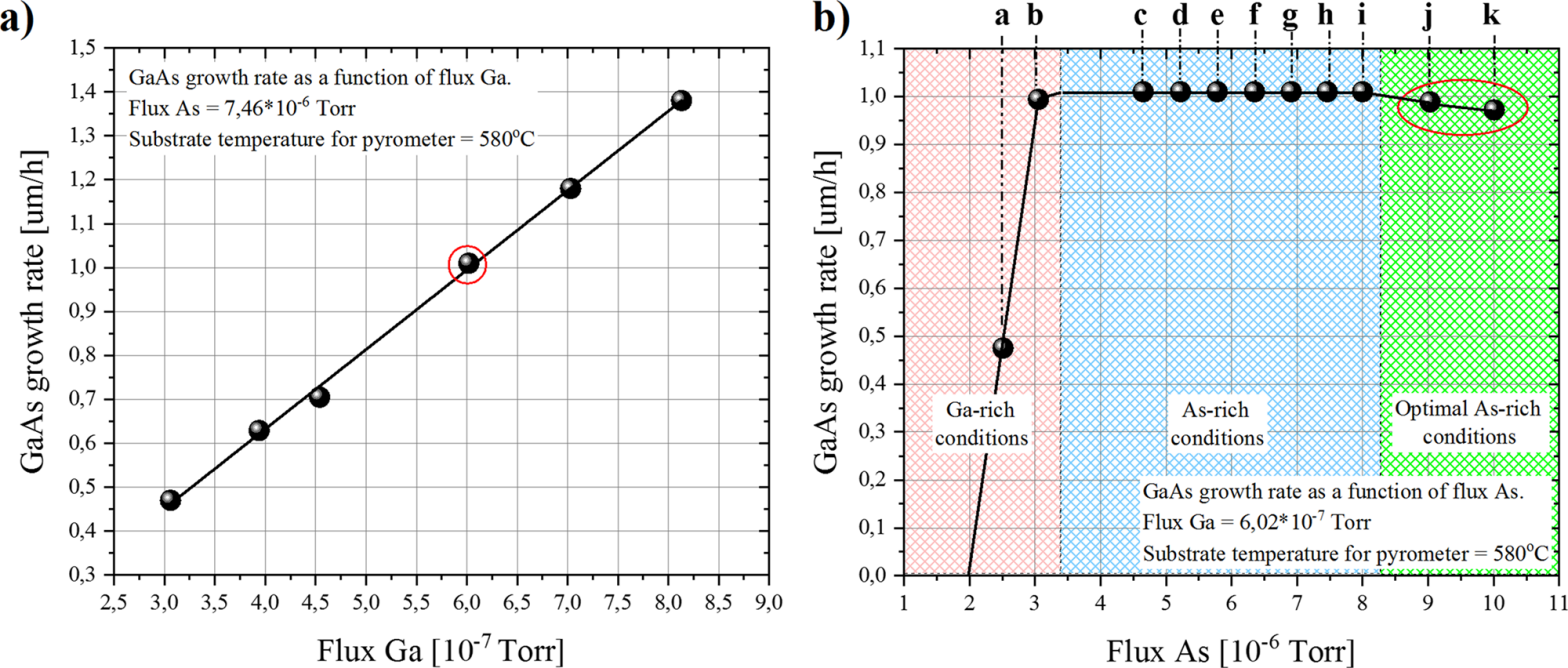
Dependence of the growth rate of GaAs layers on the gallium flux for the constant arsenic flux 7.46 × 10^–6^ Torr and the substrate temperature of 580 °C and the dependence of the growth rate of GaAs layers on the arsenic flux for the constant gallium flux 6.02 × 10^–7^ Torr and the substrate temperature of 580 °C marked as (a) and (b), respectively.

The samples used to plot GaAs growth rate dependence on gallium flux were obtained at the same growth temperature 580 °C and arsenic flux 7.46 × 10^–6^ Torr, and all GaAs layers were grown for 60 min. The growth rates were determined by post process thickness measurements achieved with a profilometer, and the values are presented in Figure 4a.

The red circled point represents a gallium flux at 6.02 × 10^–7^ Torr, for which the growth rate at 1 μm/h was obtained, which is the most preferable and used as a starting point in the next set of processes. Another conclusion possible to make at this point is that 7.46 × 10^–6^ Torr of arsenic flux is more than enough for arsenic rich growth conditions, which was confirmed with obtained growth rates above and below 1 μm/h.

### 3.3 As/Ga Flux Ratio and Growth Conditions

**Figure 5:**
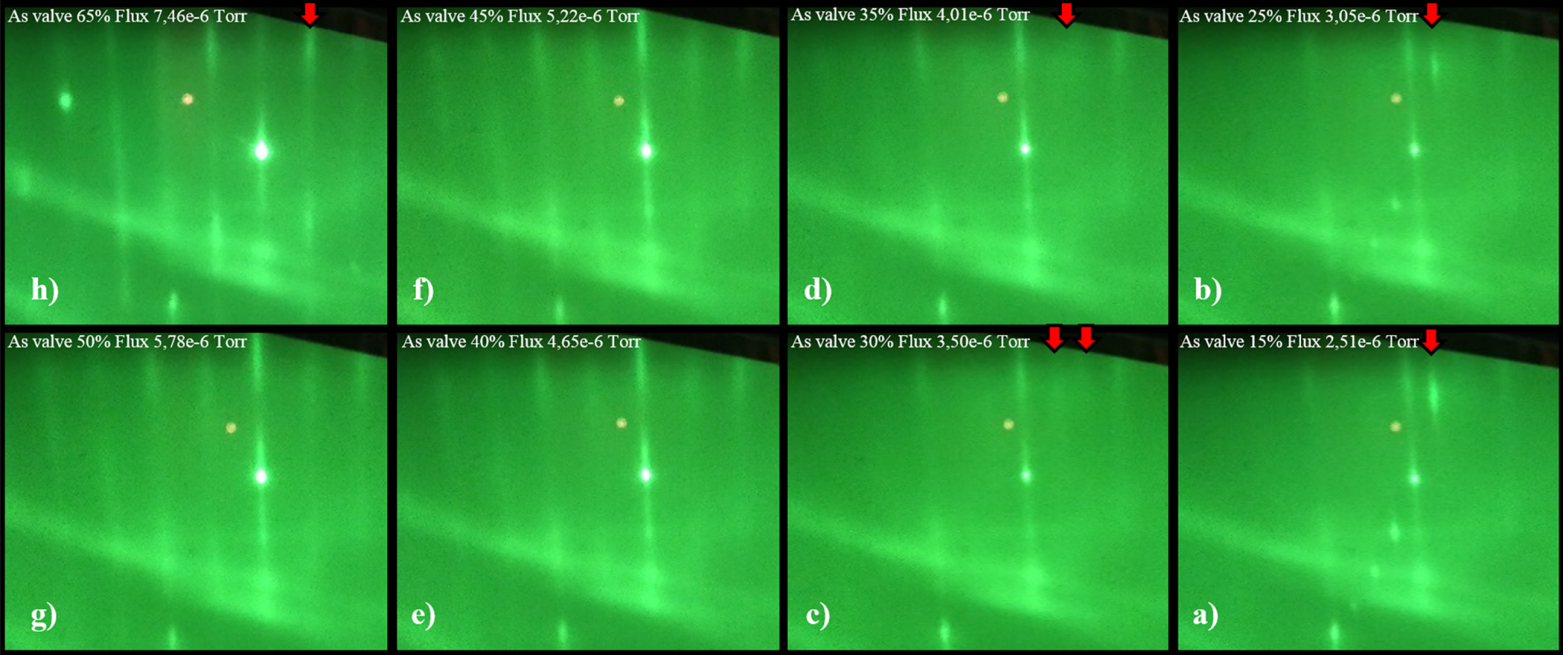
RHEED patterns during the GaAs layer growth process for smaller and smaller arsenic fluxes, respectively. Red arrows indicate where changes were observed in the RHEED pattern from GaAs azimuth [0–11].

The optimization of growth parameters of homoepitaxial GaAs layers has been conducted in the following way, the flux of arsenic element was gradually lowered at a constant Ga flux value during the growth process until the change of the RHEED pattern was observed. The pattern change from 2 × 4 to 4 × 2 reconstruction streaks pointed to the transition from arsenic-rich conditions to gallium-rich conditions. As depicted in Figure 5, the occurrence of blurring appeared (Figure 5d) after reducing As flux from the value of 7.46 × 10^–6^ Torr to 4.0 × 10^–6^ Torr, while before reaching the aforementioned flux value, the 2 × 4 reconstruction was clearly visible (Figure 5a).

**Figure 6:**
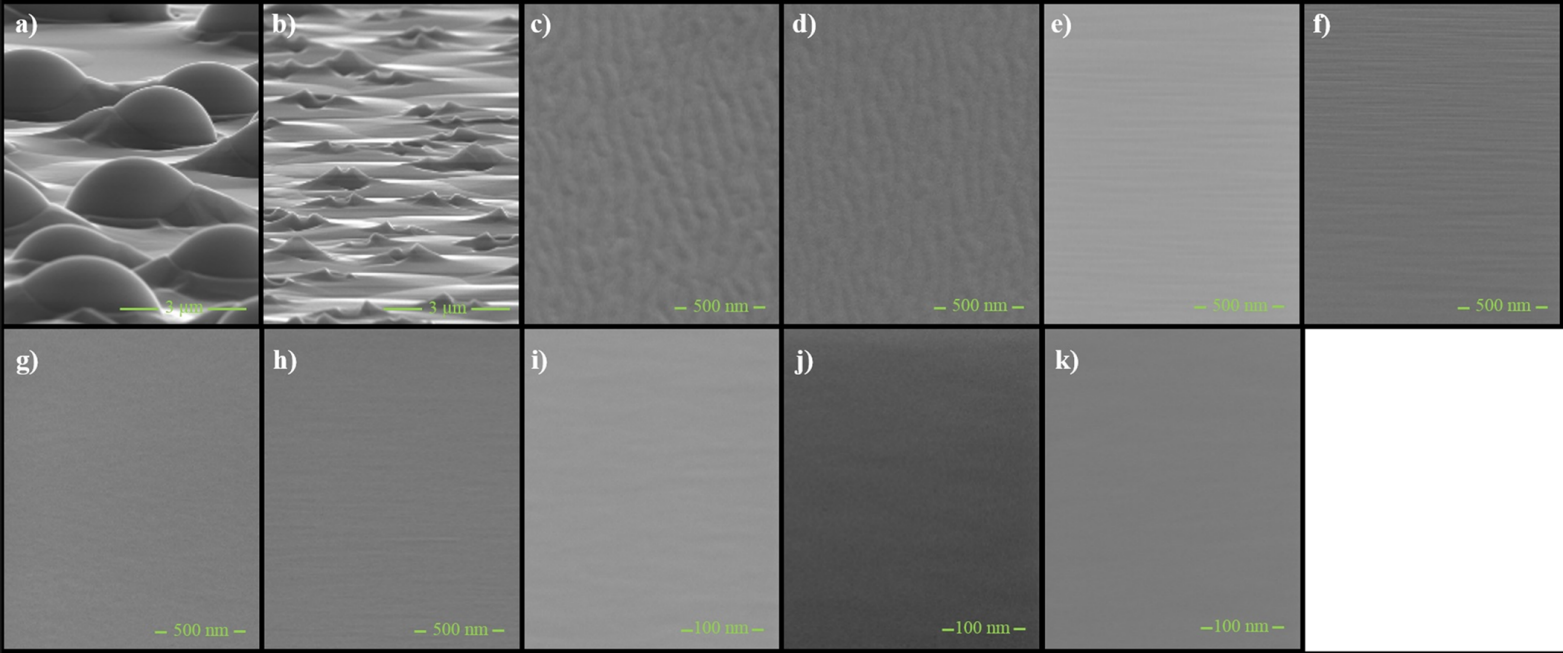
SEM images of GaAs layers inclined by 9°, increased in increasingly larger arsenic fluxes, amounting to, respectively: (a) As valve 15% Flux As = 2.51 × 10^–6^ Torr, (b) As valve 25% Flux As = 3.05 × 10^–6^ Torr, (c) As valve 40% Flux As = 4.65 × 10^–6^ Torr, (d) As valve 45% Flux As = 5.22 × 10^–6^ Torr, (e) As valve 50% Flux As = 5.78 × 10^–6^ Torr, (f) As valve 55% Flux As = 6.35 × 10^–6^ Torr, (g) As valve 60% Flux As = 6.91 × 10^–6^ Torr, (h) As valve 65% Flux As = 7.46 × 10^–6^ Torr, (i) As valve 70% Flux As = 8,00 × 10^–6^ Torr, (j) As valve 80% Flux As = 9.03 × 10^–6^ Torr, (k) As valve 90% Flux As = 1.01 × 10^–5^ Torr. The scale in the SEM images was selected according to the observed surface morphology.

Further lowering the As flux led to splitting at 3.5 × 10^–6^ Torr, which was followed with clear 4 × 2 reconstruction at the final stage (Figure 5a,b). The next step of growth parameters included obtaining a set of thin layers at a constant gallium 6.02 × 10^–7^ Torr flux and constant substrate temperature 580 °C. The symbols in Figure 4b, 6, and 7a–k corresponds to following growth processes. The change of arsenic-rich to gallium-rich conditions is evidenced by the change of the growth rate. On the other hand, the decrease of the growth rate of GaAs layers due to the increase of the arsenic flux to values in the range 9 to 10 × 10^–6^ Torr is an indication of establishment of the optimal growth conditions for deposited homo-epitaxial GaAs layers. Figure 4b represents the growth rate of GaAs as a function of As flux for constant Ga flux and substrate temperature, in detail. There are three main areas marked in Figure 4b for gallium-rich (red), arsenic-rich (blue), and optimum arsenic-rich (green) growth conditions of homoepitaxial GaAs layers, respectively, for readers’ convenience. Figure 6 contains SEM images, which presents the top view except for samples depicted in Figure 6a,b (these two cases are inclined by 9° in accordance with the full side view) of the surface of each obtained sample at aforementioned growth conditions.

**Figure 7:**
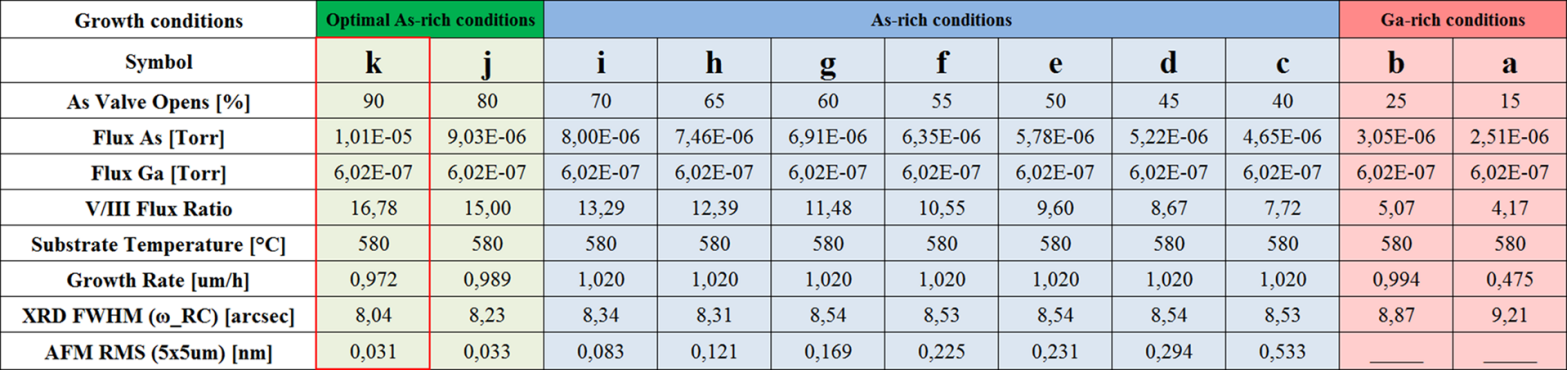
Summary of GaAs layer growth parameters with different arsenic to gallium flux ratio for the temperature gradient on the gallium effusion cell amounting to 100 °C. The table also contains surface roughness values obtained from the AMF measurements from the 5 × 5 μm area and the values of the half-width for the peak from the GaAs layer obtained from the XRD measurements in the ω-RC scan.

As it is easily noticeable, the surfaces of samples evaporated in the gallium-rich conditions are three dimensional, covered with irregular bubble shaped gnarls (Figure 6a), in which the size and coverage of the surface decrease with increase of As flux (Figure 6b). The surface reconstruction completely changes when the growth parameters moved to As-rich conditions (Figure 6c, 2nd area in the Figure 4b), for which all gnarls are eliminated and average roughness is about RMS = 0.5 nm. Further increase of As flux to about 8 × 10^–6^ Torr was highly beneficial to surface quality as the roughness decreased to about RMS = 0.08 nm. Finally, establishing optimal As-rich growth conditions allowed for the RMS value to be dropped down to about RMS = 0.03 nm (Figure 6k). All growth parameters, full width at half maxima obtained with XRD, and AFM measured RMS values for each growth process are gathered in Figure 7, for reader convenience.

A clear trend in the improvement of the surface smoothness as well as the FWHM of the GaAs peak (obtained with ω-RC scan) can be noticed, which follows an increase of As flux from process to process. A sufficiently high arsenic flux subtly changes the coefficient of adhesion of gallium atoms due to forcing the diffusion of gallium atoms on the surface of the layer and incorporation mainly along the atomic terraces, which is manifested by a slight decrease in the growth rate. The slight decrease of the GaAs layer growth rate by 3% in comparison to the highest growth rate value, together with increase of crystalline quality (see Figure 4b symbol *k* and Figure 7 symbol *k*) for which RMS and FWHM reached the lowest values, suggested to establishment of the optimal growth conditions. Summarizing this part, it should be stressed out that precise selection and control of the growth parameters resulted in obtaining vertically smooth homo-epitaxial GaAs layers.

### 3.4 Substrate Temperature and Temperature Gradient of the Gallium Effusion Cell

**Figure 8:**
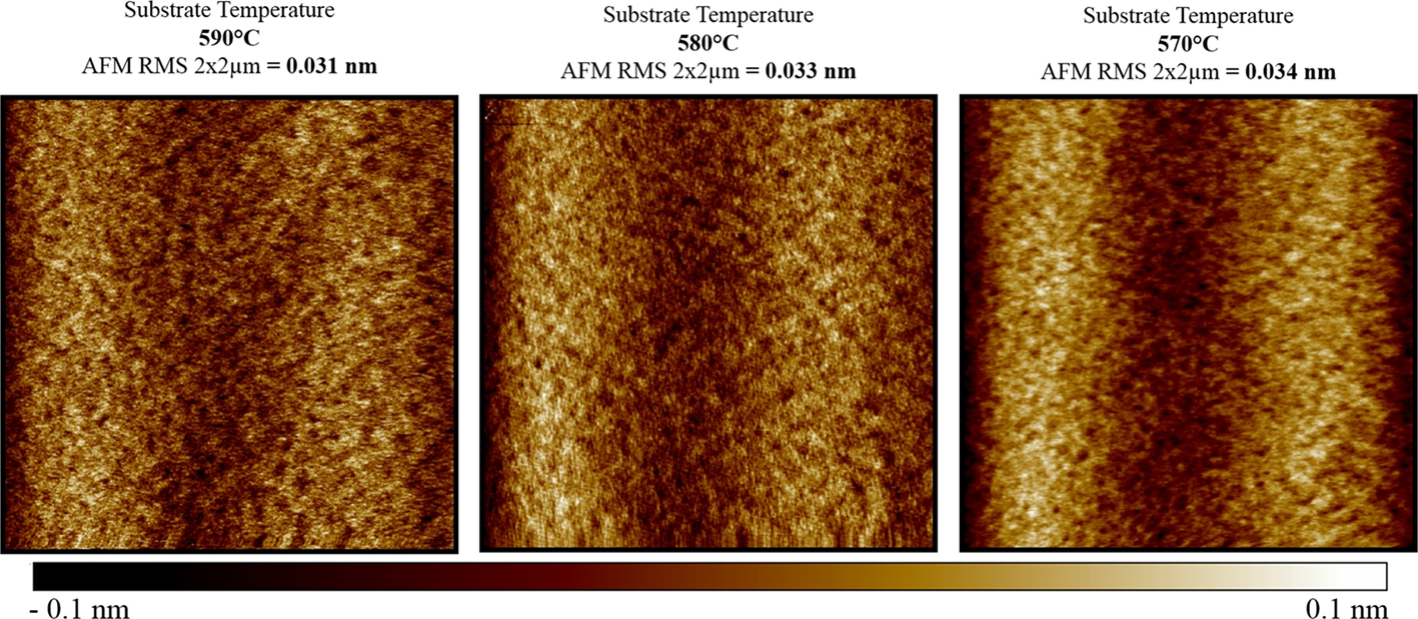
AFM images of the surface morphology of GaAs layers were increased at three different substrate temperatures.

The established growth conditions in the previous optimization stage seem to be satisfactory; therefore the next optimization stage was limited to investigation of the influence of substrate temperature on the surface morphology. Three processes were conducted for optimal growth parameters and the temperature change was in the range of 20 °C, for which *T*_s_ = 580 °C was considered as a base temperature. The AFM images depicting a resulting surface smoothness for area 2 × 2 um are presented in Figure 8.

**Figure 9:**
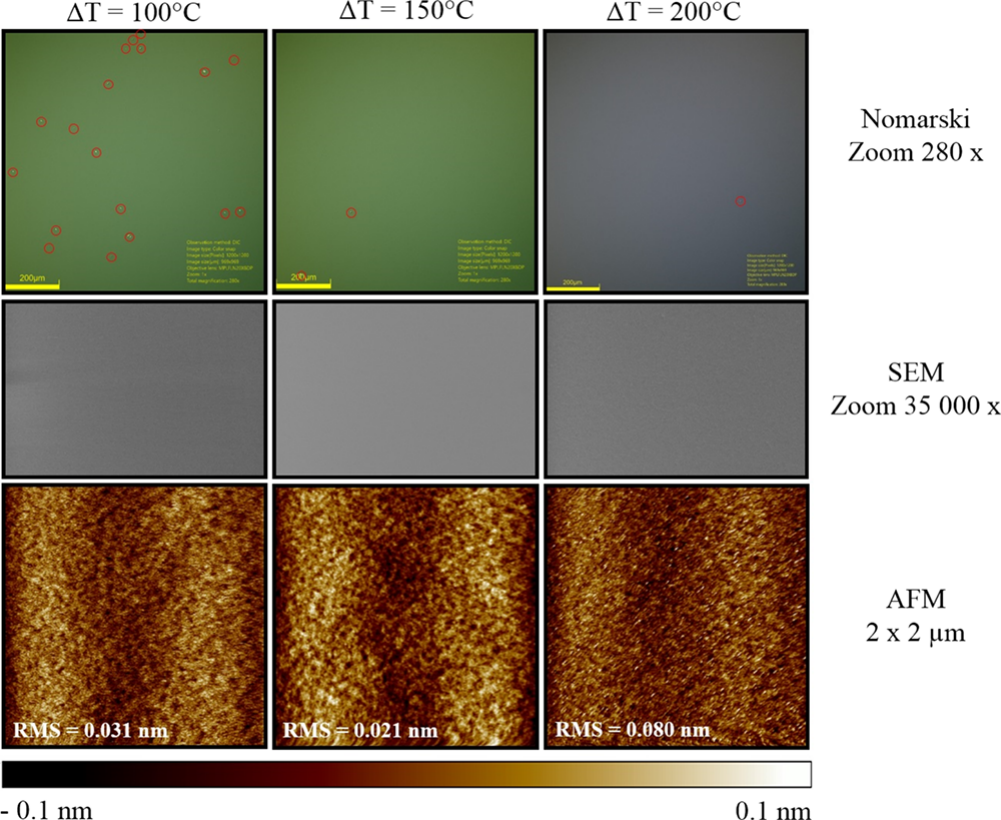
Summary of GaAs layer surface representations obtained by: Optical microscope equipped with Nomarski contrast for an area of about 1 mm^2^ (line 1). SEM surface tilted 9°, magnification 35,000× (line 2). AFM from the 2x2 μm area with the RMS roughness value plotted (line 3). The temperature gradient on the gallium effusion cell, respectively 100, 150, and 200 °C for columns 1, 2, and 3. Gallium defects on the surface of GaAs layers visible in line 1 are marked with red circles to underline them.

The measured RMS values vary slightly and may be considered as negligible; however, *T*_s_ = 590 °C was chosen for further experimentations. The results of characterization obtained with AMF and SEM techniques seem to be satisfactory, but disadvantage of alluded methods is small area of probing. The investigation of larger area of samples’ surfaces was conducted with utilization of differential interference contrast (DIC) microscopy. DIC microscopy due to the principle of interferometry of gaining information about the optical path length of the sample allows to see otherwise invisible features. For this purpose, an optical microscope equipped with Nomarski contrast optical system was used. Figure 9 top row contains images obtained with Nomarski technique, which reveal numerous (total 18) point defects on the very surface of the samples (first image to the left). The probed area was about 1 × 1 mm. The reason for presence of those defects was seen in the insufficient temperature gradient of the gallium effusion cell. As it can be noticed in the next images, the increase of the temperature gradient between bottom and top Ga ABN60 effusion cell heaters to about 150–200 °C eradicated most of the surface defects. Figure 9 allows also for direct comparison between all three characterization techniques utilized so far and exposes disadvantages of AMF and SEM techniques. Another gain at this stage of optimization was further reduction of the surface roughness to about 0.02 nm for *T*_grad_ = 150 °C at the Ga effusion cell. Unfortunately, increase of *T*_grad_ to 200 °C, although reduced the number of defects to 1 per 1 mm^2^, also increased the value of RMS to 0.08 nm. The growth parameters in terms of the best crystalline quality were established to be *T*_s_ = 590 and 150 °C of the temperature gradient of the gallium effusion cell. A possible answer to the question why increasing the temperature gradient on the Ga cell allows the reduction in the number of gallium surface defects was brought only by comparing gallium crucibles from two growth campaigns, as shown in Figure 11. During operation of the Ga cell, Ga droplets condense in the upper part of the crucible and may fall into the crucible causing material splashing and local random flux increase, which results in the formation of gallium defects on the surface of the GaAs layer. Increasing the temperature gradient on the gallium cell reduces the diameter of the condensing droplets and thus lowers the probability of material splashing out. On the other hand, increasing the temperature gradient on the gallium cell changes the properties of the gallium flux, e.g., for temperature gradients: 100, 150, and 200 °C, while maintaining the same value of the Ga flux and other growth parameters, we obtain the growth rate respectively: 0.965, 0.948, and 0.937 nm/h which suggests that the amount of gallium is decreasing and we are leaving the optimal growth conditions.

### 3.5 GaAs Layers Doped with Be and GaTe

**Figure 10:**
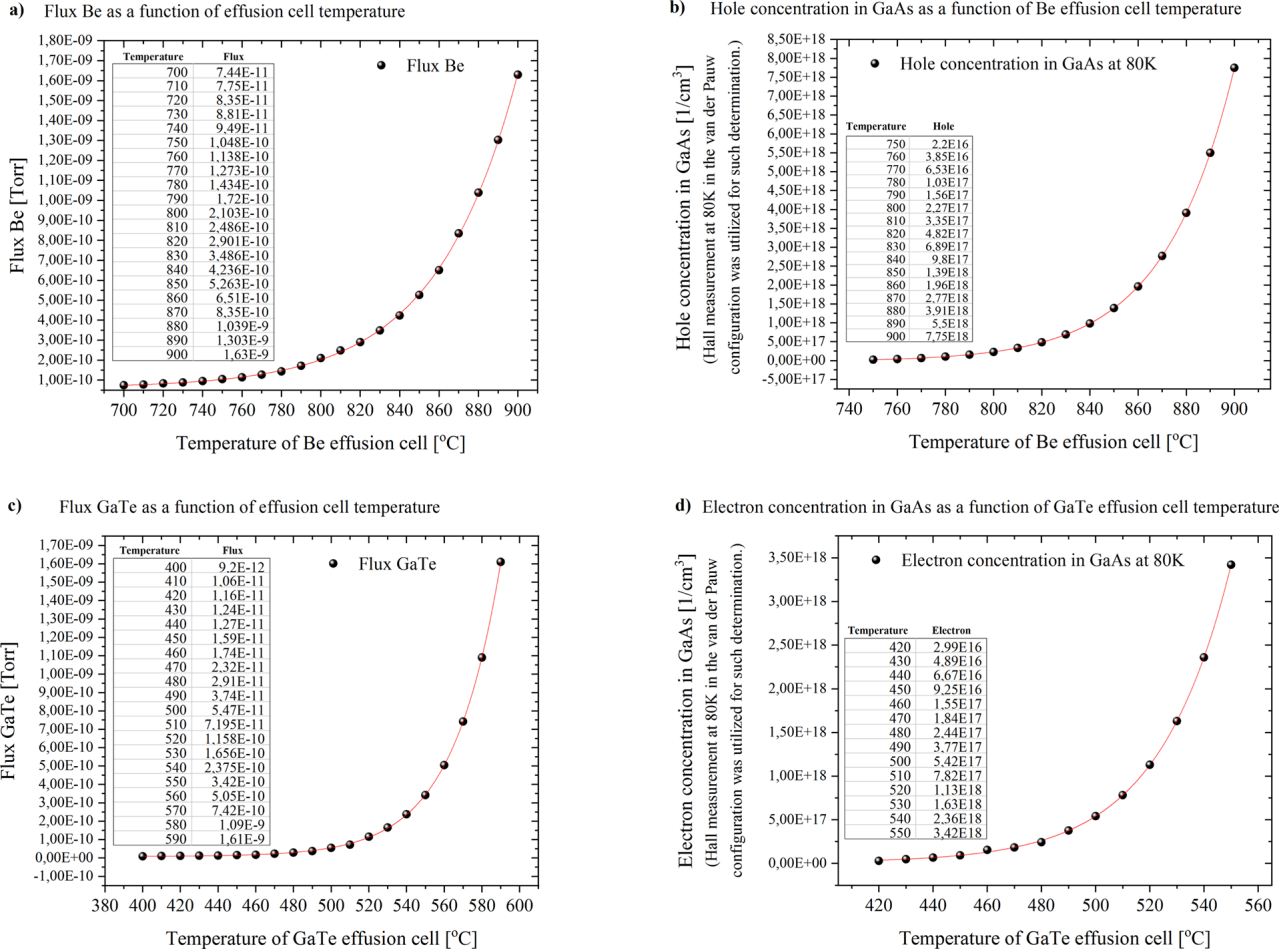
(a) Dependence of the Be flux on the effusion cell temperature. (b) Dependence of hole concentration in GaAs layers on the Be effusion cell temperature. (c) Dependence of the GaTe flux on the effusion cell temperature. (d) Dependence of electron concentration in GaAs layers on the GaTe effusion cell temperature.

Obtaining electro-optically active GaAs layers for developing industry applicable material, it is necessary to achieve p- and n-type junction in the form of a diode. Doping elements of Be or GaTe to GaAs layers enhances the concentration of holes or electrons, respectively, in the compound, making possible to achieve the alluded goal. Some attempts of doping were made for established optimal growth conditions of GaAs layers. The dependence of the acceptor or donor concentrations was determined as a function of the temperature of the dopant effusion cell. Comparing diagrams depicted in Figure 10, it may be noticed a direct correlation between temperature of Be effusion cell and flux, and also hole concentration in the GaAs layer.

In the case of undoped GaAs layer, the determined carrier concentration at 80 K was at level of 5.2 × 10^13^/cm^3^, and 3.3 × 10^14^/cm^3^ at room (300 K) temperature. Hall measurement in the van der Pauw configuration was utilized for such determination. Hole concentration at the same (80 K) temperature for Be-doped GaAs was established to be 5 orders of magnitude higher at 900 °C of Be effusion cell temperature. The same investigation was conducted for GaTe dopant as an enhancer of electron concentration in the GaAs matrix. The electron concentration for 550 °C of GaTe effusion cell temperature also increased 5 orders of magnitude in comparison to undoped GaAs layer. Figure 10d depicts exponential correlation between electron concentration and GaTe effusion cell temperature, which reaches its maximum at 550 °C. Be and GaTe fluxes were so small that their measurement required a special approach. Prior to the flux measurement, the main reactor chamber was pumped out for 24 h with the cryopanel flooded, the cells set in the standby mode, and the B–A gauge introduced. The background level was recorded (1.5 × 10^–10^ Torr) and subtracted from subsequent B–A gauge readings. The dopped cell was heated to the maximum value, and after 1 h of stabilization, the shutter was opened. The cell temperature was lowered stepwise, waiting 30 min before reading B–A gauge.

### 3.6 Comparison of Growth Campaigns 2021 and 2022

**Figure 11:**
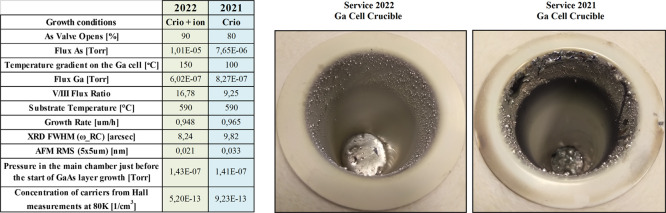
Comparison of optimal growth conditions for homoepitaxial GaAs layers from two campaigns 2021 and 2022. In addition, photographs of gallium crucibles after each campaign are included.

The growth campaign is the period between the maintenance of the growth chamber, in which optimal growth parameters are established. The changes from the previous growth parameters may be considerably large or only require a fine tune. The extent of deviation strictly depends on the scope of required maintenance. In the given case, two campaigns are considered, in which the growth parameters are considerably changed due to the addition of a new vacuum ion pump. The increase in pumping effectiveness was noticeable especially 24 h after filling of the cryopanel with liquid nitrogen. For the 2021 campaign, during which only CRYO-TORR 8 pump was working, the residual pressure level was 3.2 × 10^–9^ Torr, while during the 2022 campaign, in which two pumps were working parallelly (CRYO-TORR 8 and PI400TTZ), the residual pressure level dropped down and was two times lower 1.5 × 10^–9^ Torr. The differences in the starting parameters for the growth chamber in the standby mode were negligible, and residual pressure was comparable on the level of 1.5–1.6 × 10^–10^ Torr. The residual pressure in the main chamber right before the growth process of GaAs layers was comparable for both campaigns, but the fluxes of arsenic were disparate. For the 2022 campaign, with two vacuum pumps active, the arsenic flux was about 31% higher in comparison to the 2021 campaign. In the case of gallium flux, it was quite the opposite, the 2022 campaign required only 73% of gallium flux which was optimal in the 2021 campaign. To summarize, the additional vacuum pump drastically influenced the V/III elements ratio, which was 9.25 in 2021, and 16.78 in 2022, for reaching the most optimal growth parameters on GaAs layers. The list of optimal growth parameters for both campaigns is presented in Figure 11. The GaAs layers grown in 2021 were obtained with a temperature gradient of 100 °C on the gallium effusion cell, which resulted in a considerably high density of surface gallium defects. This downside was greatly reduced during the 2022 growth campaign, which was a direct outcome of the experiment described above, especially the permanent change of temperature gradient in the gallium effusion cell was increased to 150 °C. The effect of the change is directly visible in the effusion cell picture (Figure 11). The radius decrease of condensing gallium droplets resulted in drastic—one order of magnitude—reduction of surface gallium defects. The more effective pumping subsystem also positively influenced the pureness of obtained GaAs layers, which was confirmed with Hall effect measurement. Especially, the decrease of charge concentration at 80 K point on it.

## 4 Conclusions

In this work, we presented a step-by-step procedure for optimizing growth conditions for homo-epitaxial undoped GaAs layers and doped with Be or GaTe for p- or n-type conductivity. A clear trend was evidenced as a way to improve the crystallographic quality and surface morphology in the successive steps. The dependence of the concentration of the Be and GaTe acceptor or donor on the temperature of effusion cells was also presented. Additionally, concentration of charges from Be and GaTe dopants fluxes were calculated, which may be a reference point for the reader. We compared the optimal growth conditions for GaAs layers from both campaigns and showed the significant effect of increasing pumping efficiency on them.
